# Loss of CDH16 expression is a strong independent predictor for lymph node metastasis in Middle Eastern papillary thyroid cancer

**DOI:** 10.1038/s41598-023-45882-x

**Published:** 2023-10-29

**Authors:** Abdul K. Siraj, Sandeep Kumar Parvathareddy, Maha Al-Rasheed, Padmanaban Annaiyappanaidu, Nabil Siraj, Maximilian Lennartz, Saif S. Al-Sobhi, Fouad Al-Dayel, Guido Sauter, Khawla S. Al-Kuraya

**Affiliations:** 1 Human Cancer Genomic Research, Research Centre, King Faisal Specialist Hospital and Research Centre, MBC#98-16, P.O. Box 3354, 11211 Riyadh, Saudi Arabia; 2 Institute of Pathology, University Medical Center Hamburg-Eppendorf, Hamburg, Germany; 3 Department of Surgery, King Faisal Specialist Hospital and Research Centre, Riyadh, Saudi Arabia; 4 Department of Pathology, King Faisal Specialist Hospital and Research Centre, P.O. Box 3354, 11211 Riyadh, Saudi Arabia

**Keywords:** Biomarkers, Oncology

## Abstract

Papillary Thyroid Cancer (PTC) is the most common type of thyroid cancer. The membrane-associated glycoprotein cadherin-16 (CDH16) plays a significant role in the embryonal development of thyroid follicles and cell adhesion. Previous studies have indicated a substantial downregulation of CDH16 in PTC. However, its role in Middle Eastern PTC has not been elucidated. We analyzed a tissue microarray comprising 1606 PTC and 240 normal thyroid tissues using immunohistochemistry to assess CDH16 expression and determine its clinico-pathological associations. We also conducted *BRAF* and *TERT* mutations analyses through Sanger sequencing. Disease-free survival (DFS) was assessed using Kaplan–Meier curves. CDH16 immunostaining was seen in 100% of normal thyroid tissues but only in 9.4% of PTC tissues (*p* < 0.0001). The loss of CDH16 expression was associated with aggressive PTC characteristics including bilaterality, multifocality, extrathyroidal extension, tall cell variant, lymph node metastasis (LNM) and distant metastasis. Additionally a correlation between loss of CDH16 expression and *BRAF* and *TERT* mutations was identified. Intriguingly, upon conducting multivariate logistic regression analysis, CDH16 was determined to be an independent predictor for LNM (Odds ratio = 2.46; 95% confidence interval = 1.60–3.79; *p* < 0.0001). Furthermore, CDH16 loss was associated with a shorter DFS (*p* = 0.0015). However, when we further subdivided CDH16 negative patients based on the co-existence of *TERT* and/or *BRAF* mutations, we found that patients with both CDH16 negative expression and *TERT* mutation exhibited the shortest DFS (*p* < 0.0001). In conclusion, our results suggest that CDH16 protein expression could serve as a valuable diagnostic tool for PTC. Furthermore, these findings demonstrate that the loss of CDH16 expression is an independent predictor of LNM and may contribute to the aggressiveness of PTC. Therefore, downregulation of CDH16 in PTC might be a potential target for designing novel therapeutic strategies to treat PTC.

## Introduction

Thyroid Cancer (TC) stands as the most prevalent endocrine malignancy^1,2^, with papillary thyroid cancer (PTC) comprising over 70% of all TC cases^3,4^. Over recent years, the global incidence of TC has seen a significant upsruge^5–7^. In Saudi Arabia, TC ranks as the second most prevalent cancer, following closely behind Breast Cancer^8^. While PTC is traditionally considered an indolent form of cancer, with a promising prognosis and favorable overall survival, it is worth noting that as many as 30% of TC patients face an unfavorable clinical course , marked by local recurrence, that necessitates additional medical and/or surgical interventions^9–11^. One of the paramount risk factors contributing to local recurrence is the presence of lymph node metastases (LNM)^12,13^. Therefore, identification of predictive markers for LNM holds immense importance in preventing recurrences and providing clinicians with valuable guidance when making therapeutic and follow-up decisions for PTC patients.

Cadherin-16 (CDH16) belongs to the Cadherin superfamily, which encompasses various calcium-dependent membrane-associated glycoproteins^14^. CDH16 serves a pivotal role in maintaining cell adhesion, embryonal development and cell growth^14,15^. Its crucial involvement in mediating cell–cell adhesion has led to several studies highlighting a strong association between abnormal CDH expression and tumor progression and metastasis^16–18^. A recent study has shed light on the prognostic significance of CDH16 in renal cell carcinoma, where a reduction in CDH16 expression was identified as a potent predictor of poor prognosis^19^. Additionally, CDH16 has been implicated in the development and differentiation of thyroid gland during embryogenesis^20,21^. Interestingly, our recent study, conducted in a smaller cohort encompassing multiple organ sites revealed a lower frequency of CDH16 expression in PTC compared to normal thyroid, follicular adenomas and follicular carcinomas^22^. RNA expression studies have also suggested a significant downregulation of CDH16 in PTC in comparison to thyroiditis^23^. Utilizing The Cancer Genome Atlas (TCGA) cohort and bioinformatics analysis, they showed an association between CDH16 expression and unfavorable clinico-pathological features in TC. Moreover, a recent investigation elucidated the mechanistic role of CDH16 in TC, where CDH16 overexpression inhibited cell proliferation and migration, while inducing apoptosis, affirming its role as tumor suppressor^24^. Furthermore, by analyzing a small cohort of 35 PTC patients, they demonstrated a significant correlation between low expression of CDH16 and tumor size, stage and LNM.

Despite these intriguing prior findings, research on the immunohistochemical expression of CDH16 has predominantly focused on renal cell carcinomas^25–32^, with only one recent study in PTC^22^. Additionally, publicly accessible RNA databases have indicated CDH16 expression in cervical, endometrial and ovarian cancers^33–35^. Nevertheless, comprehensive studies involving substantial cohorts of PTC patients remain scarce. As a result, this study was specifically designed to investigate the expression of CDH16 and its potential utility as a prognostic and diagnostic marker. We conducted our study using a cohort comprising 240 normal thyroid tissues and 1606 PTC samples by immunohistochemistry. Furthermore, we delved into the clinico-pathological and molecular correlations associated with CDH16 expression in Middle Eastern PTC cases.

## Results

### Patient and tumor characteristics

Median age of the study population was 38.0 years (range: 5.9–87.6 years), with a male to female ratio of 1:3. The majority of tumors were classical variant of PTC (63.3%; 1017/1606). 33.0% (530/1606) of tumors were bilateral and 50.4% (809/1606) were multifocal. 41.4% (664/1606) of tumors exhibited extrathyroidal extension and 26.8% (430/1606) showed lymphovascular invasion. LNM was noted in 48.7% (782/1606) and distant metastasis in 8.9% (143/1606) of PTCs. *BRAF* mutation was noted in 54.9% (882/1606) and *TERT* mutation in 11.9% (191/1606) of PTCs (Table 1). Of the 882 cases with *BRAF* mutation, the following variants were detected: *BRAFV600E* (n = 871), *A1801G;K601E* (n = 3), *c.1799_1801delTGA:p.V600Efs* (n = 2), *c.1799_1801het_delTGA:p.V600fs* (n = 2), *A1844G;G615E* (n = 1), *1799_1822_insAGGGGATTTTGGTCTGGCTACAGA* (n = 1), *c.1460_1473delTGACAGCACCTACAC:p.V487fs* (n = 1) and *c.1801A* > *G:p.K601E* (n = 1). Among the cases with *TERT* mutation (n = 191), *C228T* was present in 165 cases, *C250T* in 24 cases, *C228A* in one case and combined *C228T* and *C250T* being present in one case.

**Table 1:** Clinico-pathological associations of CDH16 expression in papillary thyroid cancer.

	Total		CDH16 negative		CDH16 positive		*p* value
	No	%	No	%	No	%	
No. of patients	1606		1455	90.6	151	9.4	
Age (years)							
Median (range)	38.0 (5.9–87.6)		38.2 (5.9–87.6)		37.2 (8.5–74.2)		0.4884
≤ 55	1307	81.4	1181	81.8	126	83.4	
> 55	299	18.6	174	18.2	25	16.6	
Age group							
Pediatric/adolescent (≤ 18 years)	90	5.6	84	5.8	6	4.0	0.4581
Adult (> 18 years)	1516	94.4	1371	94.2	145	96.0	
Sex							
Female	1219	75.9	1109	76.2	110	72.8	0.3622
Male	387	24.1	346	23.8	41	27.2	
Histology type							
Classical variant	1017	63.3	940	64.6	77	51.0	< 0.0001
Follicular variant	282	17.6	232	16.0	50	33.1	
Tall-cell variant	168	10.5	162	11.1	6	4.0	
Other variants	139	8.6	121	8.3	18	11.9	
Tumor laterality							
Unilateral	1076	67.0	962	66.1	114	75.5	0.0169
Bilateral	530	33.0	493	33.9	37	24.5	
Tumor focality							
Unifocal	797	49.6	709	48.7	88	58.3	0.0252
Multifocal	809	50.4	746	51.3	63	41.7	
Extrathyroidal extension							
Absent	942	58.6	821	56.4	121	80.1	< 0.0001
Present	664	41.4	634	43.6	30	19.9	
Lymphovascular invasion							
Absent	1176	73.2	1062	73.0	114	75.5	0.5042
Present	430	26.8	393	27.0	37	24.5	
pT							
T1	640	39.8	580	40.0	60	39.7	0.4985
T2	510	31.8	464	32.0	46	30.5	
T3	332	20.7	295	20.3	37	24.5	
T4	120	7.5	112	7.7	8	5.3	
Unknown	4	0.2					
Lymph node metastasis							
Absent	679	42.3	584	44.1	95	69.8	< 0.0001
Present	782	48.7	741	55.9	41	30.2	
Unknown	145	9.0					
Distant metastasis							
Absent	1463	91.1	1318	90.6	145	96.0	0.0138
Present	143	8.9	137	9.4	6	4.0	
Stage							
I	1354	84.3	1219	84.2	135	90.0	0.1860
II	168	10.5	157	10.8	11	7.4	
III	24	1.5	22	1.5	2	1.3	
IV	53	3.3	51	3.5	2	1.3	
Unknown	7	0.4					
*BRAF* mutation							
Present	882	54.9	853	59.9	29	19.2	< 0.0001
Absent	692	43.1	570	40.1	122	80.8	
Unknown	32	2.0					
*TERT* mutation							
Present	191	11.9	179	15.3	12	8.2	0.0211
Absent	1129	70.3	994	84.7	135	91.8	
Unknown	286	17.8					
Disease-free survival time (months), median (range)	38 (5–265)		38 (5–267)		40 (6–246)		

Statistical analyses were performed only on available data and unknown cases were excluded.

### CDH16 expression and its association with clinico-pathological characteristics

**Figure 1:**
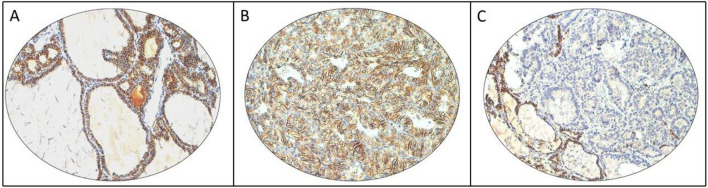
Examples of CDH16 immunostaining in normal thyroid and papillary Thyroid cancer (PTC). (**A**) Representative section of normal thyroid tissue showing strong positive membrane expression of CDH16 in the follicular cells. (**B**) PTC tissue showing positive staining for CDH16 and (**C**) another PTC tissue showing negative staining for CDH16 with adjacent normal tissue showing positive expression. (20 X/0.70 objective on an Olympus BX 51 microscope (Olympus America Inc, Center Valley, PA, USA)).

CDH16 immunostaining was predominantly membranous and was noted in 100% of normal thyroid tissues (Fig. 1A). In PTCs, CDH16 staining was noted in only 9.4% (151/1606) of cases (Fig. 1B,C). Loss of CDH16 immunostaining was significantly associated with adverse clinico-pathological characteristics such as tall-cell variant (*p* < 0.0001), bilateral tumors (*p* = 0.0169), multifocality (*p* = 0.0252), extrathyroidal extension (*p* < 0.0001), LNM (*p* < 0.0001) and distant metastasis (*p* = 0.0138) (Table 1). Interestingly, we also found a significant association between CDH16 loss and *BRAF* (*p* < 0.0001) as well as *TERT* (*p* = 0.0211) mutations (Table 1).

We further analyzed the CDH16 expression in pediatric/adolescent patients (≤ 18 years) in our cohort. Patients aged ≤ 18 years constituted 5.6% (90/1606) of the total cases. CDH16 expression was noted in 6.7% (6/90) of pediatric/adolescent cases. However, the difference in CDH16 expression between pediatric/adolescent and adult PTC was not statistically significant (*p* = 0.4581) (Table 1).

### Loss of CDH16 expression is an independent predictor of lymph node metastasis

Since LNM is an important prognostic marker in PTC, we sought to further analyze the association between CDH16 expression and LNM using logistic regression analyses. Univariate analysis revealed male sex (Odds ratio (OR) = 1.41; 95% confidence interval (CI) = 1.11–1.80; *p* = 0.0055), bilaterality (OR 2.11; 95% CI 1.68–2.64; *p* < 0.0001), multifocality (OR 1.71; 95% CI 1.39–2.10; *p* < 0.0001), extrathyroidal extension (OR 3.91; 95% CI 3.13–4.89; *p* < 0.0001), lymphovascular invasion (OR 1.86; 95% CI 1.46–2.39; *p* < 0.0001), T status (OR 1.62; 95% CI 1.27–2.06; *p* < 0.0001), distant metastasis (OR 2.44; 95% CI 1.45–4.13; *p* = 0.0008) and CDH16 loss (OR 2.94; 95% CI 2.01–4.31; *p* < 0.0001) were significant predictors of LNM. Multivariate analysis using these parameters indicated that only bilaterality (OR 1.74; 95% CI 1.23–2.45; *p* = 0.0016), extrathyroidal extension (OR 3.11; 95% CI 2.43–3.96; *p* < 0.0001) and CDH16 loss (OR 2.46; 95% CI 1.60–3.79; *p* < 0.0001) were significant independent predictors of LNM (Table 2).

**Table 2:** Logistic regression analysis for prediction of lymph node metastasis risk.

Risk factor	Univariate analysis		Multivariate analysis	
	OR (95% CI)	*P* value	OR (95% CI)	*P* value
Age (> 55 years)	0.90 (0.69–1.18)	0.4439		
Male sex	1.41 (1.11–1.80)	0.0055	1.21 (0.92–1.59)	0.1805
Aggressive Histotypes	1.12 (0.86–1.46)	0.3982		
Bilateral tumors	2.11 (1.68–2.64)	< 0.0001	1.74 (1.23–2.45)	0.0016
Multifocal tumors	1.71 (1.39–2.10)	< 0.0001	0.99 (0.72–1.36)	0.9406
Extrathyroidal extension	3.91 (3.13–4.89)	< 0.0001	3.11 (2.43–3.96)	< 0.0001
Lymphovascular invasion	1.86 (1.46–2.39)	< 0.0001	1.33 (0.99–1.78)	0.0502
T status (T3/4 vs T1/2)	1.62 (1.27–2.06)	< 0.0001	1.14 (0.87–1.49)	0.3338
Distant metastasis	2.44 (1.45–4.13)	0.0008	1.39 (0.79–2.45)	0.2542
CDH16 negative	2.94 (2.01–4.31)	< 0.0001	2.46 (1.60–3.79)	< 0.0001

*OR* odds ratio, *CI* confidence interval.

### Disease-free survival

**Figure 2:**
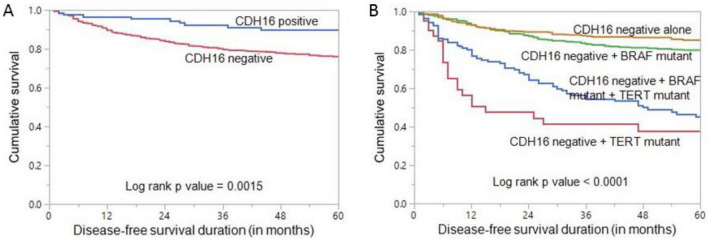
Disease-free survival (DFS). (**A**) Kaplan Meier survival plot showing statistically significant shorter DFS in CDH16 negative cases compared to CDH16 positive (*p* = 0.0015) (**B**) Kaplan Meier survival plot showing statistically significant shorter DFS in patients with co-existing CDH16 negative and *TERT* mutation (*p* < 0.0001), compared to other sub-groups.

Loss of CDH16 expression was found to be associated with significantly shorter disease-free survival (DFS) (*p* = 0.0015, Fig. 2A). Given the significant association of *BRAF* and *TERT* mutations with CDH16, we further analyzed the effect of these mutations on CDH16 loss of expression in predicting DFS. We divided the patients with CDH16 loss into four categories: CDH16 negative alone, CDH16 negative + *BRAF* mutant, CDH16 negative + *TERT* mutant and CDH16 negative + *BRAF* mutant + *TERT* mutant. Interestingly, we found that CDH16 negative + *TERT* mutant patients had the worst DFS among the four groups (*p* < 0.0001, Fig. 2B).

## Discussion

In this study, we observed a significant downregulation of CDH16 expression in PTC when compared to normal thyroid tissue. This loss of CDH16 was found to be associated with aggressive clinico-pathological characteristics and shorter DFS. The multivariate logistic regression analysis further confirmed that CDH16 stands as an independent predictor for LNM. Interestingly, when we sub-grouped CDH16 negative patients based on the presence of co-existing *TERT* and/or *BRAF* mutations, we discovered that patients with co-existing CDH16 negative expression and *TERT* mutation exhibited the shortest DFS.

In a recent study conducted by Lennartz et al.^22^, which analyzed over 10,000 tumors spanning more than 100 entities, CDH16 positivity was identified in 100% of normal thyroid tissues, 86% of 94 follicular adenomas, 60% of 67 follicular carcinomas and only 6.6% of 212 papillary thyroid carcinomas. In this study, a collection of tissue microarrays containing 1846 samples from 1606 PTC and 240 normal thyroid tissues revealed CDH16 membranous immunostaining in 100% of normal thyroid tissues, but only 9.4% of PTCs. Our finding regarding the low CDH16 positivity rate in PTC is in concordance with the results of Lennartz et al.^22^. However, it is worth noting that another study by Yang et al.^24^ reported CDH16 positivity in 51.4% of PTCs, albeit in a much smaller sample size of 35 cases. The presence of CHD16 expression in 100% of normal thyroid tissues is consistent with previous research, indicating that CDH16 expression is associated with the fully differentiated state of thyroid cells and plays a role in thyroid follicular polarity^20,21,36^. The striking absence of CDH16 expression in over 90% of PTC cases suggests a relevant clinical practical utility of using CDH16 immunostaining as a useful diagnostic tool for the identification of these tumors.

Our analysis, encompassing a substantial cohort of over 1600 PTCs, enabled us to investigate in depth the correlation between the loss of CDH16 expression and various clinico-pathological and molecular parameters. Although the loss of CDH16 expression was unrelated to age, gender, tumor size and disease stage, it exhibited strong associations with several other aggressive features like tall cell variant, extrathyroidal extension, multifocality and bilateral tumors. Most notably, we observed a significant association between the absence of CDH16 and lymph node metastasis (LNM), as well as distant metastasis. Subsequent logistic regression analyses showed that downregulated expression of CDH16 was an independent predictor of LNM. Based on the identified role of CDH16 as cell adhesion molecule that preserves tissue integrity and inhibits cell migration and invasion^24^, the increased level of aggressiveness in PTC with the loss of CDH16 expression might be driven by lower degree of cells organization and higher tumor cell ability of motility and migration. Our findings are in concordance with results from previous studies that have described reduced CDH16 expression in association with aggressive PTC phenotype^23,24^. Both Li et al.^23^ (mRNA expression) and Yang et al.^24^ (protein expression) found a significant association between the downregulation of CDH16 and LNM, with Li et al. further demonstrating that reduced expression of CDH16 was an independent predictor of LNM, consistent with our study. These findings suggest that loss of CDH16 could be associated with PTC progression. We were particularly intrigued by the association of CDH16 expression loss and *BRAF* as well as *TERT* mutation. Previous studies have shown an interaction between CDH16 and other cancer genes^23,37^. In this cohort, lack of CDH16 expression was significantly associated with poor DFS in univariate analysis using the Kaplan–Meier curve. Since both *BRAF* and *TERT* mutations are known to be aggressive markers for PTC^38–42^ and both have been shown to be associated with poor DFS, we asked whether this survival effect of CDH16 loss of expression was affected by coexisting mutations in *TERT* and *BRAF* gene mutations. Specifically, we compared the DFS of PTC patients with coexisting *TERT* and *BRAF* mutations to those with a lack of CDH16 and found that PTC patients with coexisting lack of CDH16 and the presence of *TERT* mutation had the worst DFS. This is not surprising, given the clinical and functional role of *TERT* in PTC, which we and others have shown previously^38,43,44^. *TERT* is known to promote cancer progression, leading to the loss of epithelial cell adhesion molecule, E-cadherin and induction of several mesenchymal markers such as N-cadherin and vimentin^38,45,46^. The synergistic effect of *TERT* and CDH16 on cellular adhesion and cancer progression may explain the worse DFS when *TERT* mutation and CDH16 loss coexist.

Despite these interesting findings and the large cohort included in this study, this research still has some limitations. First, this is retrospective single institute cohort from Middle Eastern ethnicity where selection bias cannot be excluded. Second, the functional mechanism between CDH16 and other genes are mainly theoretical and should be further investigated and validated. Thirdly, the results of our findings cannot be generalized to the global population and hence, future large scale studies in other ethnicities is recommended.

In conclusion, our study has revealed that CDH16 protein expression is massively downregulated in Middle Eastern PTC when compared to normal tissue, indicating its potential as a valuable diagnostic tool for identification of these tumors. Furthermore, we have provided crucial clinical evidence that lack of CDH16 expression promotes PTC aggressiveness and serves as an independent predictor for LNM in PTC patients. Patients with both CDH16 expression loss and *TERT* mutation exhibited the worst DFS. This study underscores the potential of targeting CDH16 expression in PTC as a promising avenue for the development of therapeutic strategies to treat PTC.

## Materials and methods

### Patient selection and clinico-pathological data

One thousand six-hundred and six PTC patients diagnosed between 1988 and 2020 at King Faisal Specialist Hospital and Research Centre (Riyadh, Saudi Arabia) were included in the study. Cases were identified based on clinical history followed by fine needle aspiration cytology for confirmation. Baseline clinico-pathological data were collected from case records and has been summarized in Table 1. Staging of PTC was performed using the eighth edition of American Joint Committee on Cancer (AJCC) staging system^47^.

### Ethics declarations

Institutional Review Board of King Faisal Specialist Hospital and Research Centre provided ethical approval for the current study. Research Advisory Council (RAC) granted waiver of informed consent for use of retrospective patient case data under project RAC# 2110 031 and 2211 168. All the methods were carried out in accordance with the Declaration of Helsinki.

### *BRAF *and* TERT* mutation analysis

*BRAF* and *TERT* mutation status was assessed in our laboratory by utilizing Sanger sequencing technology and has been published by us previously^38,48,49^. *BRAF* mutation analysis was performed in 1574 cases and *TERT* mutation analysis was done in 1320 cases.

### Tissue microarray (TMA) construction and immunohistochemistry (IHC) analysis

Tissue microarray (TMA) format was utilized for immunohistochemical analysis of the PTC samples. TMA was constructed as previously described^50^. Briefly, modified semiautomatic robotic precision instrument (Beecher Instruments, Woodland, WI) was used to punch tissue cylinders with a diameter of 0.6 mm from representative tumor area of the donor tissue block and brought into the recipient paraffin block. Two 0.6-mm cores of PTC were arrayed from each case.

Tissue microarray slides were processed and stained manually as described previously^51^. Primary antibody against CDH16 (monoclonal Recombinant rabbit, MSVA-516R, MS Validated Antibodies, Hamburg, Germany) was used at a dilution of 1:200 (pH 9). The Dako Envision Plus System kit was used as the secondary detection system with 3, 30-diaminobenzidine as chromogen. All slides were counter stained with hematoxylin, dehydrated, cleared and mounted. Negative controls included omission of the primary antibody. Normal tissues of different organ system were also included in the TMA to serve as control. Only fresh cut slides were stained simultaneously to minimize the influence of slide aging and maximize reproducibility of the experiment.

Staining was scored as described previously^19^. Briefly, the percentage of CDH16 positive tumor cells was estimated and the staining intensity was semi-quantitatively assessed (0, 1+, 2+ and 3+). For statistical analyses, staining results were categorized into two groups: Negative—no staining at all; Positive—staining of any intensity.

### Follow-up and study endpoint

Patients were regularly followed by both physical examinations and imaging studies to identify tumor persistence/recurrence. The median follow-up was 7.5 years (range 1.0–30.2 years). The study end-point was DFS. Patients were grouped according to disease status, with patients considered to be disease-free in the absence of clinical, biochemical (unstimulated serum thyroglobulin (Tg) levels of < 0.2 µg/L or stimulated Tg levels of < 1 µg/L in the absence of interfering thyroglobulin antibodies (TgAb)) or radiological evidence of disease persistence or recurrence. In contrast, active disease was defined by the presence of unstimulated serum Tg levels ≥ 0.2 µg/L or stimulated Tg levels ≥ 1 µg/L; a rising or denovo appearance of TgAb; or abnormal findings on radio-imaging.

### Statistical analysis

The associations between clinico-pathological variables and CDH16 protein expression was performed using contingency table analysis and Chi square tests. Mantel-Cox log-rank test was used to evaluate DFS. Survival curves were generated using the Kaplan–Meier method. Logistic regression analysis was used for univariate and multivariate analysis. Two-sided tests were used for statistical analyses with a limit of significance defined as *p* value < 0.05. Data analyses was performed using the JMP14.0 (SAS Institute, Inc., Cary, NC) software package.

## Data Availability

All data generated or analyzed during this study are included in this published article.
